# Transcriptional responses of soybean roots to colonization with the root endophytic fungus *Piriformospora indica* reveals altered phenylpropanoid and secondary metabolism

**DOI:** 10.1038/s41598-018-26809-3

**Published:** 2018-07-06

**Authors:** Ruchika Bajaj, Yinyin Huang, Sebhat Gebrechristos, Brian Mikolajczyk, Heather Brown, Ram Prasad, Ajit Varma, Kathryn E. Bushley

**Affiliations:** 1 Department of Plant Biology, University of Minnesota, Saint Paul, MN USA; 2 Amity Institute of Microbial Technology, Amity University, Uttar Pradesh Noida, India; 3 Master of Biological Sciences Program, University of Minnesota, Saint Paul, MN USA; 4 Department of Chemical Engineering and Materials Science, University of Minnesota, Minneapolis, MN USA; 5 Department of Chemistry, University of Minnesota, Minneapolis, MN USA

## Abstract

*Piriformospora indica*, a root endophytic fungus, has been shown to enhance biomass production and confer tolerance to various abiotic and biotic stresses in many plant hosts. A growth chamber experiment of soybean (*Glycine max*) colonized by *P*. *indica* compared to uninoculated control plants showed that the fungus significantly increased shoot dry weight, nutrient content, and rhizobial biomass. RNA-Seq analyses of root tissue showed upregulation of 61 genes and downregulation of 238 genes in colonized plants. Gene Ontology (GO) enrichment analyses demonstrated that upregulated genes were most significantly enriched in GO categories related to lignin biosynthesis and regulation of iron transport and metabolism but also mapped to categories of nutrient acquisition, hormone signaling, and response to drought stress. Metabolic pathway analysis revealed upregulation of genes within the phenylpropanoid and derivative pathways such as biosynthesis of monolignol subunits, flavonoids and flavonols (luteolin and quercetin), and iron scavenging siderophores. Highly enriched downregulated GO categories included heat shock proteins involved in response to heat, high-light intensity, hydrogen peroxide, and several related to plant defense. Overall, these results suggest that soybean maintains an association with this root endosymbiotic fungus that improves plant growth and nutrient acquisition, modulates abiotic stress, and promotes synergistic interactions with rhizobia.

## Introduction

Microbial symbionts, such as arbuscular mycorrhizal fungi (AMF), have the ability to promote the growth of plants through nutrient acquisition, growth promotion, and protection against biotic and abiotic stresses. Less well known are beneficial root associates belonging to the basidiomycete order Sebacinales, a ubiquitous and ecologically diverse group of fungi that universally form endophytic, and in some cases mycorrhizal-like associations, with a wide range of plants species^1–3^. They have been shown to increase nutrient acquisition^4,5^, while also improving resistance to a variety of biotic and abiotic stresses^6^.

*Piriformospora indica*, a root endophytic Sebacinaceae, was discovered in the Thar Desert of India^7^ and colonizes a wide range of plant species, forming intracellular pear shaped chlamydospores within roots^8–10^. It primarily colonizing the outer root cortex both inter- and intracellularly and generally requires host cell death for proliferation^10^. In barley, the fungus displays a biphasic lifestyle, initially colonizing living roots as a biotroph and subsequently initiating cell death that leads to proliferation and saprotrophic growth within dead root cells^10,11^. However, unlike traditional hemibiotrophs, cell death does not appear to harm the plant host and instead colonization by the fungus confers many benefits^12^, ranging from increased aboveground growth and biomass^8,13–16^ to increased flower and fruit production^17,18^ to resistance to various abiotic (e.g. salt, water, heat stress)^19–22^ and biotic (e.g. herbivores, and microbial pathogens) stresses^13,23–25^.

The mechanisms by which *P*. *indica* confers host benefits are not fully understood. The fungus has been shown to increase uptake of nutrients such as N and P as well as several micronutrients^26–28^ and impacts many other plant metabolic processes. The production and signaling of the phytohormones ethylene, jasmonic acid (JA), gibberellic acid (GA), salicylic acid (SA) and abscisic acid (ABA) have all been shown to be impacted by *P*. *indica*^29–31^. Changes in levels of these hormones likely mediate abiotic stress responses^13,32^ as well as regulate biotic responses such as induced and systemic plant defense responses, microbe-associated molecular patterns (MAMP) triggered immunity^6,29,33^, and autoregulation of mutualistic symbionts^34–36^. However, *P*. *indica* must maintain a complex communication with its host to both allow its own colonization while also enhancing defense responses against pathogens. Studies have also revealed that fungal colonization may increase plant production of defensive secondary metabolites like artemisinin^37–39^, bacoside^40,41^, forskolin^18,42^, volatile oils such as curcumin^43^, and lignans such as the anticancer podophyllotoxins that may derive from the phenylpropanoid pathway^44–47^. The phenylpropanoid pathway also provides precursors for many other plant secondary metabolite pathways, including synthesis of flavonoids and coumarins, among others^48^, and is often induced by plants in response to pathogens.

The beneficial effects of *P*. *indica* have been extensively researched using the model plant *Arabidopsis thaliana* and a number of important crop species including barley, wheat, and corn^19,49–52^, as well as angiosperms such as tomato^17^. While many studies have used molecular genetic approaches to investigate targeted sets of genes, genome scale transcriptional approaches to characterize host responses have only been conducted in *Arabidopsis* and barley^29,53^. Research investigating interactions between *P*. *indica* and legume species has been limited to a handful of studies on soybean^25,54^ and a few other legume hosts^55,56^. Soybean (*Glycine max* (L.) Merr.) is the most widely planted oil crop and is also a valuable source of protein in many parts of the world. Soybean is also known to form root symbioses with other microbes such as AMF and rhizobia, which also have been demonstrated to enhance tolerance against various abiotic and biotic stresses^57,58^. Here we report the first whole genome-scale transcriptional study of *P*. *indica* colonizing a legume host. Our results show many of the same plant growth responses observed in *Arabidopsis* and barley but also uncover responses unique to soybean, including potentially synergistic interactions with rhizobial symbionts. Our results also support findings that a mechanism of programmed cell death (PCD) distinct from the plant hypersensitivity response (HR) is involved in the symbiosis.

## Results

### Plant Growth Response and Nutrient Status

**Figure 1:**
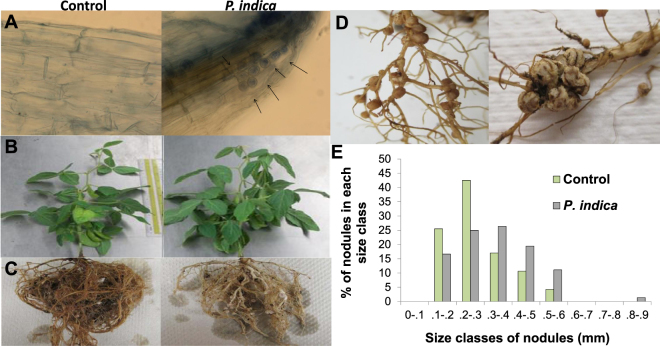
(**A**) Roots of control and *P*. *indica* treatments as seen under 40X magnification after staining with lactophenol cotton blue. Pear-shaped chlamydospores were observed in *P*. *indica* treatment (arrows) only while no chlamydospores was observed in the control. (**B**) Shoots of control and *P*. *indica* colonized plants. There was no significant difference in shoot lengths but the shoot dry weights increased in *P*. *indica* treatment. (**C**) Roots of control and *P*. *indica* colonized plants. The root dry weight decreased in *P*. *indica* colonized plants. (**D**) Nodules in control and *P*. *indica* treatments. Numbers of nodules decreased while average size increased in *P*. *indica* treatment.

Soybean roots showed 41.47% colonization at 60 days after seeding in the *P*. *indica* inoculated plants, whereas the controls showed no colonization (Fig. 1A and Supplementary Table S1. Diagnostic pear-shaped spores of *P*. *indica*^7^ were observed under the microscope and chlamydospores were found as isolated spores, pairs, tetrads, long chains, or clusters (Fig. 1A). The shoot:root ratio and shoot dry weight both increased significantly as well as numbers of leaves (5.21%), flowers (27.23%), and pods (13.14%) at 60 days after seeding (Table 1). In contrast, the dry weights and volumes of roots decreased by 8.17% and 31.25%, respectively (Fig. 1C and Table 1). Thickening of both the primary and lateral roots was observed in the *P*. *indica* treatment (Fig. 1C). Colonization with *P*. *indica* also impacted nodule formation by rhizobia. While the number of nodules decreased slightly in the *P*. *indica* treatment (70.66 ± 12.42) compared to the control (96.33 ± 10.21), the ratio of dry weight nodules: dry weight roots (0.100 ± 0.013) was significantly larger than in the controls (0.043 ± 0.024 mm) (Table 1). The average diameter of nodules in the P. indica treatment was also observed to be slightly larger (treatment (0.29 ± 0.03 mm) than the control treatment (0.25 ± 0.04 mm), although this difference was not statistically significant (Fig. 1D, Table 1). A histogram of size classes of nodules showed that a larger proportion of nodules in the P. indica treatment fell into larger size classes (0.3 - 0.4, 0.4 - 0.5, and 0.5 - 0.6 mm) than those of the control (Fig. 1D and E).

**Table 1:** Plant Growth and Nutrient Status.

	Control (C)	*P*. *indica* (Pi)	% increase of Pi/C
**Growth Parameters**			
Percent Colonization (%)	0.00 ± 0.00	41.47 ± 7.12*	41.47
Dry shoot biomass(g)	2.02 ± 0.45	2.28 ± 0.43*	12.87
Dry root biomass (g)	0.90 ± 0.21	0.73 ± 0.17*	−18.89
Root volume (mL)	10.67 ± 1.15	7.33 ± 1.57*	−31.30
Number of leaves	31.00 ± 2.65	39.67 ± 3.06*	27.96
Number of branches	11.00 ± 1.00	12.00 ± 2.65*	9.09
Number of flowers	6.00 ± 2.64	9.33 ± 2.08*	55.50
Number of pods	7.33 ± 2.08	8.00 ± 2.00	9.14
Number of nodules	96.33 ± 10.21	70.66 ± 12.42	−26.65
Dry nodule biomass (g)	0.096 ± 0.038	0.147 ± 0.093	51.99
Dry nodule biomass/dry root biomass (g)	0.043 ± 0.024	0.101 ± 0.014*	100.32
Dry nodule biomass/dry shoot biomass (g)	0.024 ± 0.009	0.050 ± 0.012*	100.04
**Nutrient Status**			
Nitrogen (%)	22.43 ± 0.13	23.71 ± 0.03*	5.77
Phosphorous (mg/kg)	1493.33 ± 55.08	1663.33 ± 160.10*	11.38
Potassium (mg/kg)	14728.00 ± 415.67	15730.33 ± 1615.09*	6.81

The results show the mean and standard deviation calculated from three plant biological replicates. An asterisk (*) indicates significance at p = 0.05 using Dunnett’s multiple comparison tests between control and *P*. *indica* treatment.

The concentration of nitrogen (N), phosphorus (P), and potassium (K) increased significantly in shoots of *P*. *indica* colonized plants (Table 1). Micronutrients, including aluminum, manganese, zinc, and nickel as well as the beneficial nutrients including calcium, magnesium, sodium, and especially iron also increased significantly in colonized plants (Supplementary Table S2). The concentrations of boron, copper, cadmium, and chromium decreased, although not significantly (Supplementary Table S2).

### RNA-Seq Analyses

**Figure 2:**
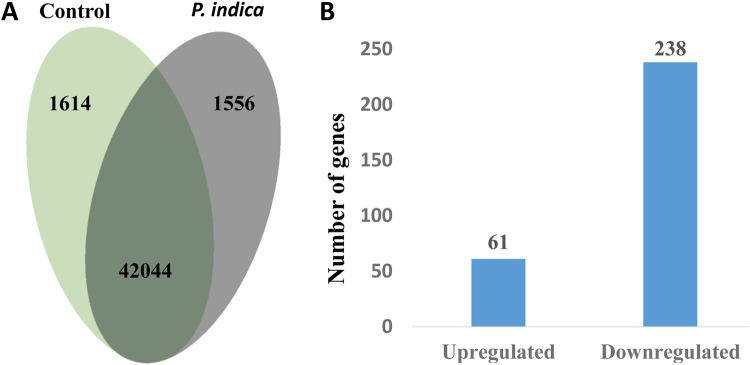
(**A**) Total number of genes expressed in control only (light green), *P*. *indica* treatment only (gray), and genes expressed in both conditions (dark green). (**B**) Graph of the number of genes upregulated and down regulated in *P*. *indica* colonized roots compared to the control at FDR = 0.05.

Over 90% of reads from each treatment aligned to the soybean Williams 82 genome a2.v1 and were evenly balanced across treatments with an average of 12 million reads per sample (Supplementary Table S3). We found that a large number of soybean genes, 42,044 out of a total of 56,044 models in Williams 82 a2.v1 annotations, or roughly 75% of annotated gene were expressed in both conditions, while 1556 were uniquely expressed in the *P*. *indica* treatment and 1614 were uniquely expressed in the control (Fig. 2). Differential expression of several genes was validated using qRT-PCR (Supplementary Table S4). In the differential expression analysis, 61 genes were significantly upregulated while 238 were downregulated (Fig. 2 and Supplementary Tables S5 and S6). Among the most highly upregulated genes, many belonged to functional categories known to be upregulated by *P*. *indica* in other crops, including hormone metabolism and nutrient acquisition (Table 2). Other categories of highly upregulated genes included lignin and cell-wall metabolism, amino acid metabolism, and sugar metabolism. (Table 2) Among downregulated genes, a majority were heat shock proteins with putative functions in abiotic stress response but also included genes involved in amino acid metabolism, cell-wall metabolism, and plant defense regulators of PCD and response to oxidative stress (Table 3).

**Table 2:** Twenty most highly upregulated genes by fold-change in *P*. *indica* treatment.

Soybean ID	Arabidopsis homolog	Log_2_ FoldChange	Description
**Amino Acid Metabolism**			
Glyma.04G020200	AT5G11880	2.38	Pyridoxal-dependent decarboxylase family protein
Glyma.03G189800	AT2G36570	3.01	Leucine-rich repeat protein kinase family protein
**Cell Wall Metabolism**			
Glyma.18G263700	AT5G54160	2.41	O-methyltransferase 1
Glyma.06G286200	AT4G35160	2.77	O-methyltransferase family protein
Glyma.01G211000	AT1G12240	2.11	Glycosyl hydrolases family 32 protein
**Hormone Metabolism**			
Glyma.18G250100	AT2G04160	2.07	Subtilisin-like serine endopeptidase family protein
Glyma.13G183600	AT1G61120	2.09	terpene synthase 04
Glyma.03G168000	AT1G15520	2.31	pleiotropic drug resistance 12
Glyma.17G092800	AT5G14920	2.38	Gibberellin-regulated family protein
Glyma.11G184800	AT2G04160	2.89	Subtilisin-like serine endopeptidase family protein
**Nutrient Acquisition**			
Glyma.12G178500	AT2G28160	2.01	FER-like regulator of iron uptake
Glyma.10G139800	AT3G62020	2.03	germin-like protein 10
Glyma.14G011800	AT5G55970	2.10	RING/U-box superfamily protein
Glyma.13G191400	AT5G07010	2.31	sulfotransferase 2 A
Glyma.07G014500	AT4G25150	2.82	HAD superfamily, subfamily IIIB acid phosphatase
Glyma.08G076000	AT3G43660	2.71	Vacuolar iron transporter (VIT) family protein
**Lipid Metabolism**			
Glyma.17G139900	AT4G12510	2.22	Bifunctional inhibitor/lipid-transfer protein/seed storage 2S albumin superfamily protein
**Sugar Metabolism**			
Glyma.03G229800	AT1G56600	2.82	galactinol synthase 2
Glyma.17G242400	AT4G25000	3.20	alpha-amylase-like
**Unknown**			
Glyma.10G224300	NA	2.39	N/A
Glyma.11G213500	AT5G37478	2.23	TPX2 (targeting protein for Xklp2) protein family
Glyma.15G014500	AT1G79960	2.22	ovate family protein 14

**Table 3:** Twenty most highly downregulated genes by fold-change in *P*. *indica* treatment.

Soybean ID	Arabidopsis homologue	Log_2_ Fold Change	Description
**Abiotic Stress Response**			
Glyma.08G212000	AT1G52560	−6.29	HSP20-like chaperones superfamily protein
Glyma.06G202200	AT1G74310	−5.95	heat shock protein 101
Glyma.04G229800	AT4G27670	−5.79	heat shock protein 21
Glyma.14G099900	AT5G12020	−4.92	17.6 kDa class II heat shock protein
Glyma.06G134900	AT4G27670	−4.91	heat shock protein 21
Glyma.10G029600	AT3G22830	−4.90	heat shock transcription factor A6B
Glyma.16G206200	AT4G10250	−4.66	HSP20-like chaperones superfamily protein
Glyma.16G012000	AT1G54050	−4.46	HSP20-like chaperones superfamily protein
Glyma.02G205600	AT1G16030	−4.45	heat shock protein 70B
Glyma.10G176400	AT4G10250	−4.78	HSP20-like chaperones superfamily protein
**Amino Acid Metabolism**			
Glyma.08G321200	AT5G10770	−6.32	Eukaryotic aspartyl protease family protein
Glyma.08G321500	AT5G10770	−4.97	Eukaryotic aspartyl protease family protein
Glyma.10G184700	AT2G38870	−4.73	Serine protease inhibitor, potato inhibitor I-type family protein
**Cell Wall Metabolism**			
Glyma.05G065700	AT4G17030	−6.77	expansin-like B1
Glyma.17G147400	AT4G17030	−6.30	expansin-like B1
Glyma.17G147500	AT4G17030	−4.51	expansin-like B1
**Plant Defense**			
Glyma.09G284700	AT4G11290	−5.11	Peroxidase superfamily protein
Glyma.07G061500	AT2G46240	−6.38	BCL-2-associated athanogene 6
**Unknown**			
Glyma.16G069500	AT5G01750	−5.80	Protein of unknown function (DUF567)
Glyma.01G036300	NA	−4.37	

### GO Term Analyses and Visualization with REVIGO

**Figure 3:**
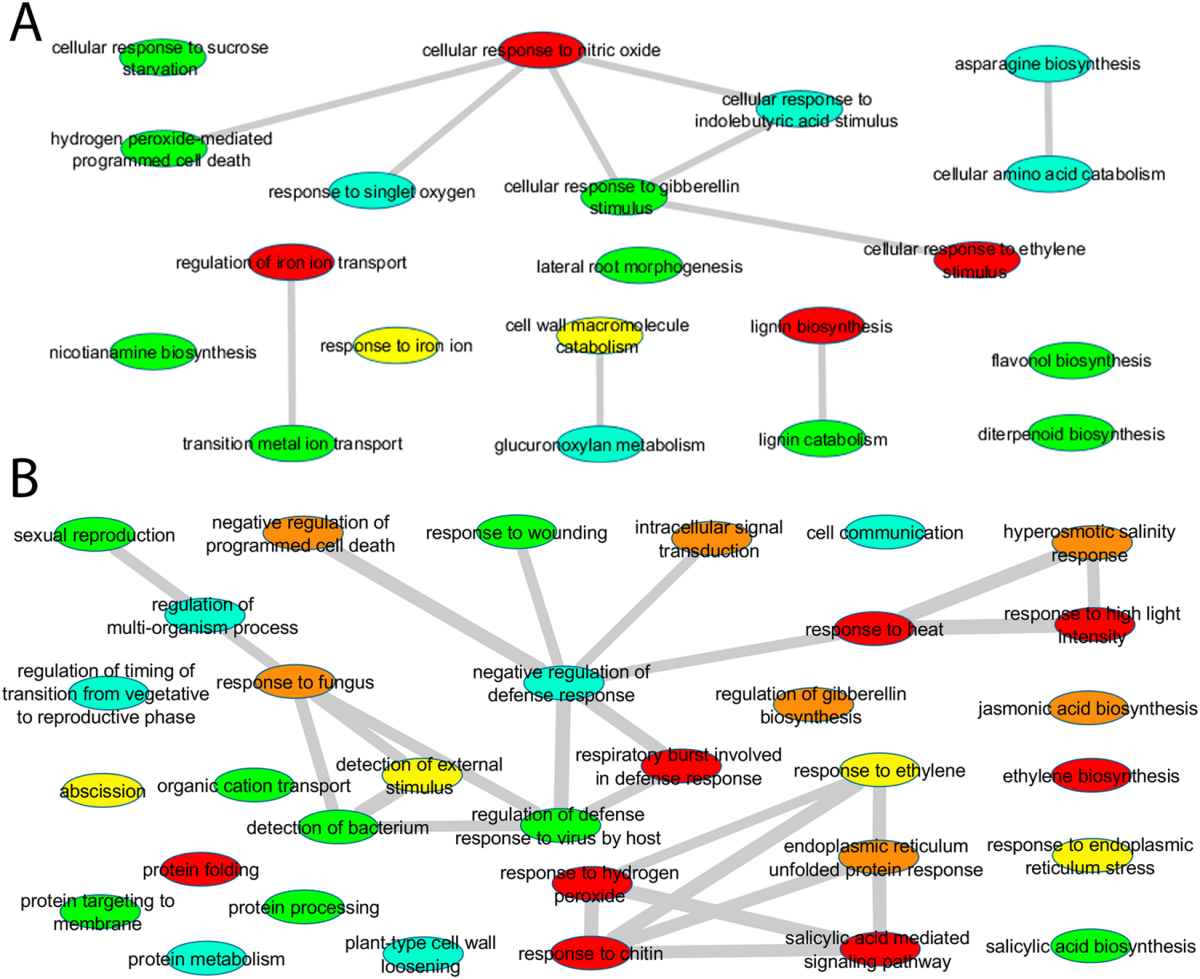
Differentially expressed genes summarized as a tree network using REVIGO. (**A**) GO terms related to biological processes of upregulated genes in the comparison of control versus *P*. *indica*. (**B**) GO terms related to biological processes of downregulated genes in the comparison control versus *P*. *indica*. GO categories are represented by circles and are color coded according to corrected p-value for GO enrichment analysis. Red indicates categories that are significantly enriched at p < 0.05 while nonsignificant categories are color coded with corrected p < 0.5 = orange, p < 1 = yellow, p < 5 = green, and p < 10 = blue). Lines indicate connections between categories within the GO hierarchy and singletons have been grouped by similar function.

GO term enrichment analyses were performed using the Soybase (http://www.soybase.org) GO enrichment tool. GO categories were displayed as tree networks using ReviGO and were visualized in Cytoscape^59–61^. Upregulated genes showed the most significant (corrected p-value < 0.005) enrichment of the biological process categories of lignin biosynthesis (GO:0009809) (Fig. 3A and Supplementary Table S7). Other significantly (corrected p-value < 0.05) enriched categories included the regulation of iron transport (GO:0034756), cellular response to nitric oxide (GO:0071732) and ethylene stimulus (GO:0071369). GO categories that included upregulated genes but were not statistically enriched included cellular response to iron ion (GO:0071281), cell wall macromolecule catabolic process (GO:0016998), transition metal ion transport (GO:0000041), diterpenoid biosynthetic process (GO:0016102), flavonol biosynthesis (GO:0051555), response to sucrose starvation (GO:0043617), and hydrogen peroxide-mediated PCD (GO:0010421).

Downregulated genes that showed the most significant (corrected p-value < 0.0001) enrichment included biological processes related to response to heat (GO:0009408), response to high light intensity (GO:0009644), and response to hydrogen peroxide (GO:0042542) (Fig. 3B and Supplementary Table S8). Heat acclimation (GO:0010286), salicylic acid mediated signaling pathway (GO:0009863), protein folding (GO:0006457), and response to chitin (GO:0010200) were also highly statistically (corrected p-value < 0.003) enriched (Fig. 3B and Supplementary Table S8). Other significantly (corrected p-value < 0.05) enriched categories included several involved in plant defense, including respiratory burst involved in defense (GO:0002679), salicylic acid signaling (GO:0009863), and response to chitin (GO:0010200) as well as hormone metabolism including ethylene biosynthetic process (GO:0009693) and abscisic acid mediated signaling pathway GO:0009738). Downregulated GO categories that were not significantly enriched included others in plant defense or pathogen recognition, such as jasmonic acid (GO:0009867) mediated signaling, negative regulation of PCD (GO:0043069), regulation of plant-type hypersensitive response (GO:0010363), regulation of gibberellin biosynthesis (GO:0010370), response to endoplasmic reticulum stress (GO:0034976), response to fungus (GO:0009620), and response to bacterium (GO:0016045) (Fig. 3B and Supplementary Table S8). Other downregulated genes belonged to GO categories involved in abiotic stress responses including hyperosmotic salinity response (GO:0042538) (Fig. 3B and Supplementary Table S8).

Overall, these enriched GO categories suggest that, in response to *P*. *indica*, the soybean plant induces a growth response, increasing expression of genes in GO categories related to lignin biosynthesis, cell wall construction and remodeling, and iron uptake while downregulating genes involved in plant defense and heat shock proteins involved in response to several abiotic stresses (heat, high light, hydrogen peroxide, and salinity).

### Metabolic Pathway Analysis

Differentially expressed genes and their associated log_2_ fold change values were mapped onto the SoyCyc metabolic pathway database. General categories of metabolism containing upregulated genes included anabolic processes involved in secondary metabolite biosynthesis, amino acid metabolism, fatty acid metabolism, hormone biosynthesis, amine and polyamine biosynthesis, and carbohydrate biosynthesis as well as catabolic processes of amino acid degradation and inorganic nutrient metabolism (Supplementary Table S9). Downregulated genes mapped to these same general categories as well as cell structure and aromatic compound biosynthesis and a number of catabolic processes such as carbohydrate, amino acid, fatty acid, secondary metabolite, amine/polyamine, and hormone degradation (Supplementary Table S9).

**Figure 4:**
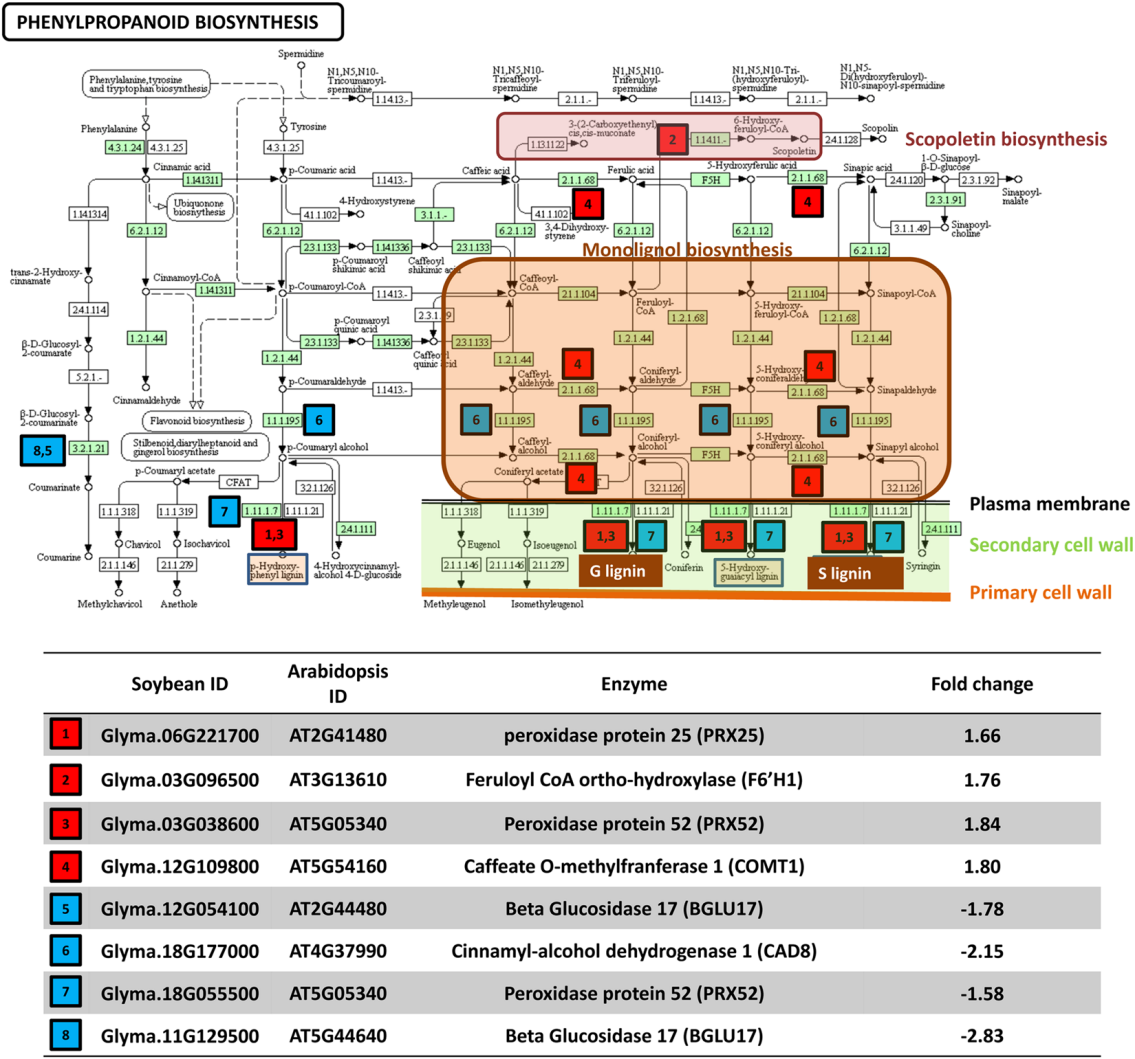
Effect of colonization with *P*. *indica* on the expression of genes within the phenylpropanoid biosynthetic pathway. Red colored squares indicate genes upregulated in the comparison of control versus *P*. *indica*; blue colored squares indicate down-regulated genes in this comparison. Homologs in *Arabidopsis* of all upregulated and downregulated soybean genes were mapped to the *Arabidopsis* KEGG pathway for phenylpropanoid biosynthesis^144–146^. Enzyme Commission (EC) codes colored in green boxes represent enzymatic steps present in soybean. Steps involved in biosynthesis of monolignol precursors sinapyl alcohol, coniferal alcohol, and 5-hydroxy-coniferal alcohol are shaded in the orange box. The final steps of production of G and S lignin (shaded brown box) are performed by peroxidases located within the plant cell wall. Biosynthesis of the iron-scavenging coumarin scopoletin is shaded in the pink box.

**Figure 5:**
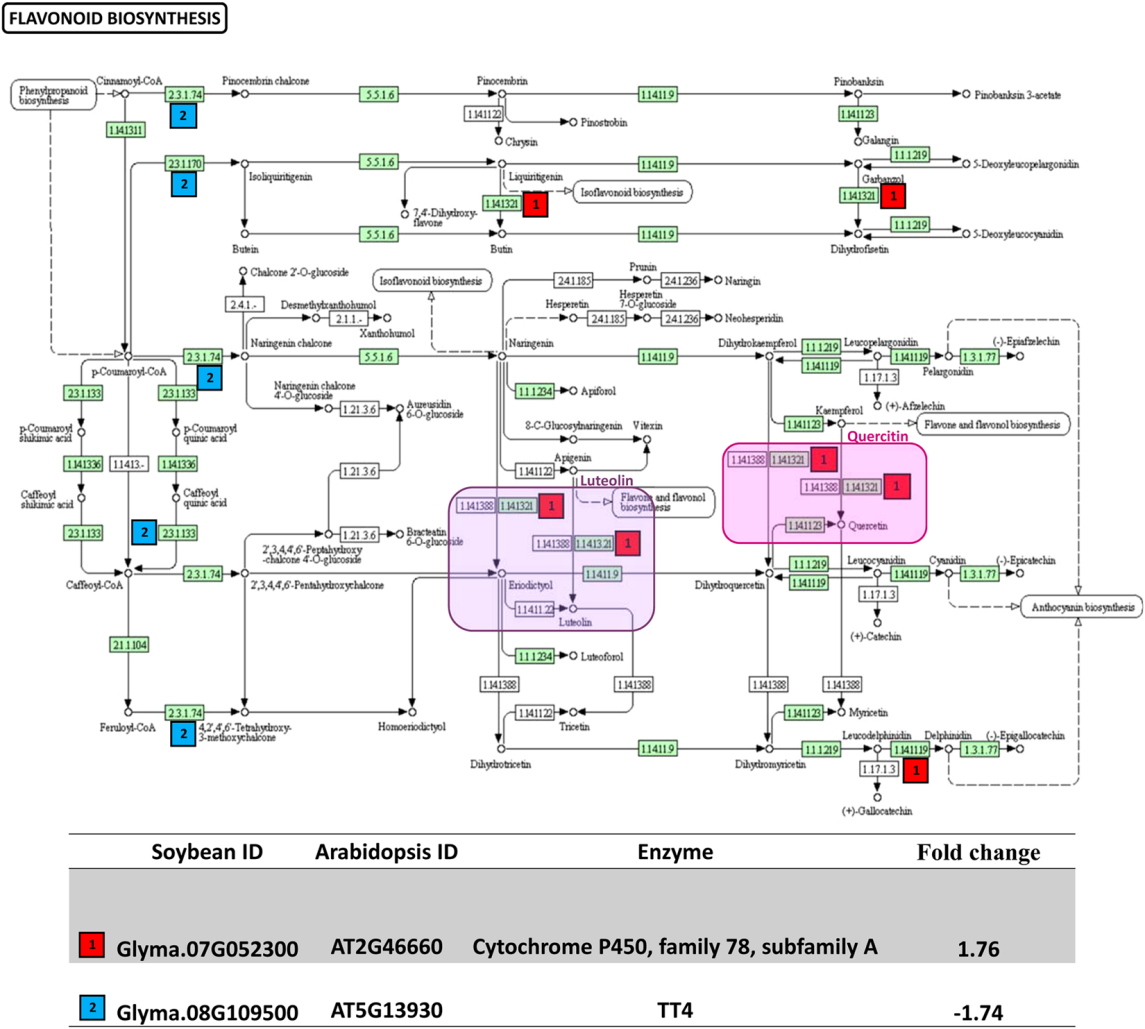
Effect of colonization with *P*. *indica* on the expression of genes within the flavonoid biosynthetic pathway. Red colored squares indicate genes upregulated in the comparison of control versus *P*. *indica*; blue colored squares indicate down-regulated genes in this comparison. Homologs in *Arabidopsis* of all upregulated and downregulated soybean genes were mapped to the *Arabidopsis* KEGG pathway for flavonoid biosynthesis^144–146^. Enzyme Commission (EC) codes colored in green boxes represent enzymatic steps present in soybean. While several steps early in the pathway are downregulated, genes in steps leading to biosynthesis of luteolin (purple box) and quercetin (pink box) are upregulated.

Among upregulated genes, several mapped to portions of the phenylpropanoid pathway responsible for production of monolignols and the iron scavenging compound scopoletin (Supplementary Table S9 and S10 and Fig. 4) as well as pathways derived from the phenylpropanoid pathway involved in biosynthesis of secondary metabolites such as flavonoids (luteolin, quercetin, leucodelphinidin, and leucocyanidin), resveratrol, phloridzin as well as furaneols and B series fagopyritols (Fig. 5 and Supplementary Tables S9 and S10). Upregulated genes also mapped to biosynthetic pathways of two terpenoid compounds, linalool and 1,3,5-trimethoxybenzene (Supplementary Table S9). Within primary metabolism, upregulated genes mapped to several fatty acid biosynthesis pathways (triacylglycerol and CDP-diacylglycerol), the carbohydrate biosynthetic pathway for stachyose, several amino acid pathways (citrulline, asparagine), and the biosynthesis of the amine/polyamine putrescine (Supplementary Tables S9 and S10). Other key pathways included inorganic nutrient metabolism, specifically phosphate acquisition through purple acid phosphatases^62^, and hormone metabolism (hydroxyjasmonate sulfate biosynthesis) (Supplementary Tables S9 and S10). Several catabolic pathways including betanidin, starch, chitin, glutamine, sucrose III, and superoxide radicals degradation also contained upregulated genes (Supplementary Tables S9 and S10).

Among downregulated genes, several also mapped to secondary metabolite pathways, including those involved in synthesis of kaemferol and dihydrokaemferol, the isoflavenoids pinkobanksin and dalochin, and a single step in the leucodelphinidin and leucocyanidin biosynthetic pathway (Fig. 5 and Supplementary Tables S9 and S10). Downregulated genes also mapped to primary metabolic anabolic pathways of fatty acid biosynthesis (very long chain fatty acids, sphingolipids, phosphatidamide, and triacylglycerol). Overall, more downregulated genes than upregulated genes mapped to catabolic pathways, including several carbohydrate (chitin, and sucrose III), amino acid (glutamine), fatty acid (triacylglycerol), secondary metabolite (betanidin, linamarin, and lotaustralin), and amine/polyamine degradation (spermidine) pathways.

### Lignin and Cell Wall Biosynthesis

Lignin biosynthesis was one of the most highly enriched GO categories among upregulated genes in colonized plants and included genes involved in monolignol biosynthesis as well as several oxidative enzymes with potential roles in polymerization of monolignols to form lignin (Fig. 4 and Supplementary Table S7). The monomer subunits of lignin, or monolignols such as H (caffeic), C (coniferal), G (guaiacyl), and S (syringin) subunits, are produced through the phenylpropanoid pathway^63^ (Fig. 4). In our data, a number of genes involved in synthesis of monolignols, including a homolog of caffeate o-methyltransferase 1 (COMT; AT5G54160; Glyma.18G263700), involved in synthesis of precursors of G (coniferyl alcohol) and S (sinapyl alcohol) lignin, were upregulated (Fig. 4 and Supplementary Tables S5 and S7). During lignification of the cell wall, monolignols are transported across the plasma membrane where oxidative enzymes, including laccases and peroxidases, catalyze oxidative polymerization of the monolignol subunits^63^. The enriched GO category for lignin biosynthesis (GO:0009809) also included two peroxidases (Glyma.06G221700 and Glyma.03G038600) whose homologs in *Arabidopsis* (AT2G41480 and AT5G05340) are involved in plant cell wall biosynthesis (Fig. 4 and Supplementary Tables S5 and S7) and two chitinase-like proteins (ATG16920; Glyma.15G143600 and Glyma.09G038500) (Supplementary Table S7). Additionally, several members of the laccase-diphenol oxidase family (Glyma.14G062300 and Glyma.03G077900), with potential roles in polymerization of monolignols, were highly upregulated (Supplementary Tables S5 and S7). A mutant of the homolog of these laccases in *Arabidopsis* (LACCASE 4; AT2G38080) has shown defects in xylem lignin deposition^64^, specifically within protoxylem^65^.

In addition to lignin metabolism, upregulated genes mapped to several metabolic pathways involved in cell wall formation and remodeling. Genes involved in biosynthesis of suberin, a lipid compound found in the cell walls of root epidermal tissues that helps reduce water loss and entry of pathogenic fungi and bacteria^66^, were upregulated (Supplementary Tables S5 and S7). Synthesis of genes involved in biosynthesis of suberin precursors, such as octodecenedioate, and in biosynthesis of cutin, however, were downregulated (Supplementary Tables S6 and S8). Other upregulated genes with potential roles in cell wall remodeling included a xyloglucan endotransglucosylase/hydrolase 32 family gene (Supplementary Tables S5 and S7).

### Flavonoid and Secondary Metabolite Biosynthesis

The phenylpropanoid pathway is also the starting point for the synthesis of many plant secondary compounds^48^. The first enzyme in the phenylpropanoid pathway, phenylalanine ammonium lyase, converts phenylalanine to p-coumaroyl CoA, a key branch-point leading to the synthesis of many different classes of plant secondary compounds including flavonoids, anthocyanins, tannins, coumarins, and lignans, among others^48^. Although a number of early steps in the flavonoid biosynthetic pathway and biosynthesis of some flavonoid compounds were downregulated, cytochrome P450 enzymes involved in production of the flavonoid compounds luteolin and quercetin were significantly upregulated (Fig. 5 and Supplementary Tables S5, S7, and S9). Upregulated genes also mapped to pathways involved in biosynthesis of aromatic and flavonoid related compounds derived from the phenylpropanoid pathway (1, 3, 5 trimethoxybenzene, furaneols) and the sugar-derived secondary metabolite B-type fagopyritols (Supplementary Table S9). Other classes of upregulated secondary metabolite genes included a terpene synthase (GES; AT1G61120; Glyma.13g183600), involved in biosynthesis of the volatile monoterpene alcohol linalool (Supplementary Tables S5 and S7).

### Nutrient Acquisition

Colonization with *P*. *indica* increased shoot content of N, P, and K (Table 1). Upregulated genes mapped to the purple acid phosphatase (PAP) pathway^62^ (Supplementary Tables S7 and S9), known to be involved in phosphorus solubilization and acquisition^67^. The homolog of an *Arabidopsis* acid phosphatase gene (AT4G25150; Glyma.07G014500) that is induced by phosphate starvation and oxidative stress conditions^68^, was upregulated in colonized plants (Supplementary Tables S5 and S7). In uptake of N, GmSAT1 (AT4G37850; Glyma.15G061400), an ammonium transporter identified as being involved in transfer of ammonium from rhizobia to plants^69^, was not differentially expressed in our data (Supplementary Tables S5 and S6). In contrast, three high affinity ammonium transporters (AT5G60770, AT2G382901, and AT2G38290; Glyma.13G323800, Glyma.07G153800 and Glyma.01G123400, respectively) were downregulated (Supplementary Tables S6 and S8). Other sources of N include urea and amino acids. One urea transporter, an aquaporin called delta tonoplast integral protein (δ-TIP) (AtTIP2; 1; AT3G16240; Glyma.07g018000), was upregulated in the *P*. *indica* treatment^70^ (Supplementary Tables S5). No genes related to K uptake were differentially expressed in our dataset.

Iron is a limiting nutrient for growth of most organisms, including plants^71,72^, and also serves as a component of many enzymes involved in lignin biosynthesis^73^. The significantly enriched GO category of iron transport **(**GO:0034756) included two FER-like regulators of iron uptake (Glyma.13g322100 and Glyma.12g178500), both homologs of the FIT1 (AT2G28160) transcription factor that controls many genes involved in iron chelation and transport (Supplementary Table S7)^74^. A homolog of nicotianamine synthase (NAS1; AT5G04950; Glyma.15g251300)^75^, responsible for biosynthesis of the chelating agent nicotianamine which transports metal ions including iron^76^, was one of the most highly upregulated genes (Supplementary Tables S5 and S10). Nicotianamine is also an intermediate in the biosynthesis of the iron scavenging siderophore 2-hydroxymugineic acid^67^ (Fig. 4). Similarly, homologs of feruloyl-CoA 6-Hydroxylase 1 (F6′H1; AT3G13610; Glyma.03g096500 and Glyma.07g124400), were upregulated (Fig. 4 and Supplementary Table S5). This gene is induced under iron deficiency and is required for the biosynthesis of fluorescent coumarins and other Fe (III) mobilizing compounds such as scopoletin, which are released into the rhizosphere for iron scavenging^77^.

### Hormone Metabolism

Upregulation of genes involved in abscisic acid, auxin, and gibberellin synthesis, signaling, or transport was observed. A membrane bound ATP binding cassette transporter that functions in the uptake of ABA into plant cells^78,79^ (ABCG40; AT1G15520; Glyma.03g168000) was upregulated (Supplementary Tables S5 and S7). Additionally, several ABA responsive genes with potential roles in drought tolerance, including a galactinol synthase (GolS2; AT1G56600; Glyma. 03g229800) and a homolog of a glycosyl hydrolase 32 (GH32) family protein sucrose vacuolar invertase (ATbetaFruct4; AT1G12240; Glyma.01g211000), were also upregulated (Supplementary Tables S5 and S7). An overexpression mutant of ATbetaFruct4 has been shown to be highly expressed in leaf stomatal guard cells and to contribute to increased drought tolerance in *Arabidopsis*^80^.

Although not significantly enriched, several upregulated genes belonged to GO categories cellular response to gibberellin stimulus (GO:0071370), gibberellic acid mediated signaling pathway (GO:0009740), and response to gibberellin stimulus (GO:0009739) (Fig. 3 and Supplementary Table S7). In addition to ATbetaFruct4 (AT1G12240; Glyma.01g211000) discussed above, these included homologs of the GA regulated gene GASA14 (AT5G14920; Glyma.17g092800) (Supplementary Tables S5 and S7). In contrast, a negative transcriptional regulator of GA biosynthesis, termed dwarf and delayed flowering phenotype (DDF1; AT1G12610; Glyma.01g216000 and Glyma.05g049900) was down-regulated (Supplementary Tables S5 and S7).

A number of auxin responsive genes with potential roles in shaping root development and architecture were also upregulated by *P*. *indica*, including two genes within the GO category lateral root morphogenesis (GO:0010102), Glyma.11g184800 and Glyma.18G250100, whose closest homolog in *Arabidopsis* is auxin-induced in root cultures 3 (AIR3; AT2G04160) (Supplementary Tables S5 and S7). AIR3 is a serine-peptidase that is localized to the cell wall and the extracellular region and is thought to have a role in remodeling of the cell wall to allow for lateral root morphogenesis^81^. An auxin transporter homologous to ABCG37 (AT3G53480; Glyma.17G034700), involved in distribution and homeostasis of auxin in roots and negative regulation of polar auxin transport inhibitors, was also upregulated (Supplementary Tables S5 and S7). ABCG37 shows broad substrate specificity for various auxin related compounds, including synthetic auxins and auxin transport inhibitors (IBA)^82–84^, and can transport caffeic acid and chlorogenic acid^85^ as well as iron scavenging compounds such as scopoletin^85^, and thus may also play a role in Fe uptake.

### Abiotic Stress Responses

Although our experiment did not expose plants to high salinity or osmotic stress, several genes induced by these stresses were observed to be upregulated in response to colonization by *P*. *indica*. In addition to homologs of the two ABA-inducible drought response genes discussed above (GolS2 and ATbetaFruct4), a homolog of the SNF1-related protein kinase (SnRK2.4; AT1G10940; Glyma.08g127000), whose overexpression showed enhanced survival rates under low water potential potentially due to their stronger ability to retain water^86^, was also upregulated (Supplementary Table S5).

Among the most highly downregulated and enriched GO categories were response to heat (GO:0009408), response to high light intensity (GO:0009644), and response to hydrogen peroxide (GO:0042542) (Fig. 3B and Supplementary Table S8). All of these categories included a large and diverse number of heat-shock family proteins and molecular chaperones as well as trypsin and other protease inhibitors (Supplementary Table S8). Several proteins involving in sensing and signaling high light or high temperature were also downregulated, including a homolog of thermosensitive gametophytic male sterility TMS1 (AT3G08970; Glyma.07g152900), which contributes to normal pollen tube development at high-temperatures (Supplementary Tables 6 and 8).

### Biotic Stress Response

A number of genes involved in the plant immune response were downregulated in response to *P*. *indica*, including several MAMP responsive genes, a homolog of AtWRKY33 (AT2G38470; Glyma.01G128100) as well as two other WRKY transcription factors, WRKY40 (AT1G80840; Glyma.13G370100) and WRKY 41 (AT4G11070; Glyma.08G021900) (Supplementary Tables S6 and S8). WRKY 40, in particular, belongs to the GO category response to fungus (GO:0009620) and is also  known to be involved in response to the hemibiotrophic bacterial pathogen *Pseudomonas syringae*^87^. Additionally, RIPK (AT2G05940; Glyma.09g069900), a positive regulator of pattern triggered immunity (PTI)^88–90^, as well as genes in the GO category response to chitin (GO:0010200), were downregulated (Supplementary Tables S6 and S8).

Genes belonging to several GO categories involved in the typical response of plants against pathogens were downregulated, including respiratory burst involved in defense response (GO:0002679), and defense response to fungus, incompatible interaction (GO:0009817) (Fig. 3B and Supplementary Tables S6 and S8). Similarly, homologs of the MLO family of seven membrane transcription factors (AT2G39200; Glyma.12G119300, Glyma.06G286800, Glyma.16G145500) involved in incompatible responses to pathogens (e.g. MLO12 for barley mildew resistance locus)^91,92^, and homologs of 2-oxoglutarate (2OG; AT3G13610; Glyma.03g096500), an Fe(II)-dependent oxygenase involved in hydrogen peroxide mediated PCD, were downregulated (Supplementary Tables S6 and S8). However, several genes in the GO category negative regulation of PCD (GO:0043069) were downregulated. Interestingly, downregulated genes also fell into several GO categories for response to endoplasmic reticulum stress (GO:0034976), endoplasmic reticulum unfolded protein response (GO:0030968), and protein folding (GO:0006457) (Fig. 3B and Supplementary Table S8).

## Discussion

Overall, soybean showed growth responses to *P*. *indica* colonization similar to those observed in other hosts, including increased shoot biomass, decreased root biomass, and increased number of flowers and pods (Table 1). The fungus also improved nutrient uptake of macronutrients N, P, and K as well as a number of micronutrients and beneficial nutrients, especially Fe (Table 1 and Supplementary Table S2). Interesting, potential synergistic interactions with rhizobia were observed with *P*. *indica*, with a greater ratio of dry weight of nodules: roots (Table 1). Although differences in average size of nodules was not significantly different, the data showed a trend of fewer but slightly larger nodules in the P. indica treatment compared to the control. Previous studies have found a strong correlation between between average nodule size and the symbiotic efficiency of nitrogen fixation and accumulation in plant tissues^93,94^.

Lignin biosynthesis, whose monolignol precursors are produced through the phenylpropanoid pathway, was one of the most highly enriched upregulated GO categories. Several other recent studies have shown that colonization with *P*. *indica* increases activity of genes within the phenylpropanoid pathway. A joint metabolomics and transcriptomics study of *P*. *indica* colonizing *Arabidopsis* detected enrichment of transcripts in the phenylpropanoid pathway as well as increased levels of oligolignol metabolites within roots^95^. However, another metabolomics study in *Arabidopsis* detected significantly lower levels of oligolignols in root exudates^53^. Together, these studies suggest that oligolignols are likely utilized within roots, potentially through transformation into lignin. Lignification and changes in cell wall metabolism are often a component of the plant defense response to strengthen cell walls against invading pathogens. The phenylpropanoid pathway has been shown to be upregulated in response to various types of pathogens including plant parasitic nematodes^96^. Interestingly, increased lignification in leaves was also observed in tropical plants colonized by horizontally transmitted endophytes^97^. We hypothesize that while endophytes may be able to evade systemic plant defenses, they may still trigger localized responses and increased lignification may occur in response to both pathogenic and endophytic fungi. Alternatively, increased lignin production could serve other specialized functions such as strengthening of xylem vessels, supported by the upregulation of a homolog of *Arabidopsis* LACCASE 4 (AT2G38080) (Supplementary Table S5), potentially contributing to the increased water use efficiency and drought tolerance observed for plants colonized by *P*. *indica*.

Previous studies have also demonstrated increased production of other secondary metabolites, including phenolic compounds derived from either the phenylpropanoid or flavonoid biosynthetic pathways. Tashackori *et al*.^46^ documented the accumulation of the toxic and anticancer lignan podophyllotoxin, as well as other phenolic defense compounds (cinnamic, coumaric, caffeic and ferulic acid), in hairy roots of *Linum album* exposed to *P*. *indica*. Volatile terpenoids have also been shown to be induced by colonization with *P*. *indica*^18,37,39,40,98,99^. In our data, upregulation of genes involved in biosynthesis of the flavonoids luteolin and quercitin (Fig. 4), as well as terpenoids, was observed (Fig. 5 and Supplementary Tables S5 and S10). Flavonoids are important secondary compounds in soybean and may play roles ranging from promoting colonization by rhizobia to protecting plants from both abiotic (UV light) and biotic stresses^100^. Luteolin, in particular, has been shown to play a key role in signaling of nodulation genes^101^ and may be one factor contributing to the larger dry-weight of nodules per dry-weight or roots observed in *P*. *indica* colonized roots (Fig. 1 and Table 1). The bioflavonoid quercetin is a powerful anti-oxidant that provides protection from oxidative stress^102,103^.

The mechanisms by which *P*. *indica* and other endophytic fungi uptake macronutrients and beneficial nutrients are poorly understood and remain controversial^104,105^. Although some studies have shown that *P*. *indica* enhances P uptake and accumulation in some hosts, others have failed to show this effect^106,107^. Our results showed significant increases in N, P, and K in plant shoot tissue, demonstrating that in soybean, the fungus improves macronutrient uptake (Table 1). Potential mechanisms of uptake and transfer could involve either fungal or plant genes or both. A high-affinity phosphate transporter (PiPT) from *P*. *indica* was previously shown to be involved in uptake and transfer of P by *P*. *indica* to *Zea mays*^27^. Other studies have shown that *P*. *indica* is able to solubilize inorganic P by lowering the pH through secretion of organic acids^108,109^. Four different fungal high-affinity phosphate transporters, as well as two acid phosphatases in *P*. *indica,* were shown to be differentially expressed in response to different concentrations of inorganic P^105^. Studies of plant responses to *P*. *indica* have shown increased expression of plant genes such as PHOSPHATE 1 (PHO1; AT3G23430.1), involved in transfer of P from root cortical cells to xylem in *Arabidopsis*^110^. Homologs of PHOSPHATE 1 were not upregulated in our data, but we show increased expression of other plant genes involved in P acquisition, including a PAP gene (Supplementary Tables S5 and S7). PAPs are upregulated in plants in response to phosphorus deficiency^67^ and during phosphorus acquisition in interactions with other symbionts including rhizobia^111^ and AMF^112^. Thus, increased solubility of plant-unavailable P in the rhizosphere through activity of both fungal and plant PAPs is likely an important factor contributing to improved P nutrition in *P. indica* colonized plants.

The effects of *P*. *indica* on N uptake have been less well characterized. The genome of *P*. *indica* contains a lower number of nitrate reductases and nitrate transporters than most fungi but contains transporters for other nitrogen sources, including a urea permease (DUR3), high-affinity ammonium transporters, and amino acid-transporters^11^. However, the fungus has been shown to induce nitrate assimilation genes in plant hosts, including an NADH-dependent nitrate reductase (AT1G37130)^26^. We did not find a homolog of this gene differentially expressed in soybean (Supplementary Tables S5 and S6). We speculate that *P*. *indica* may either utilize its own, to date uncharacterized, ammonium, urea, or amino acid transporter for uptake and transfer of N to the plant or may stimulate rhizobial symbionts. In the plant, we observed downregulation of several plant high-affinity ammonium transporters (Table 1 and Supplementary Table S6), but upregulation of one urea transporter (AtTIP2; 1; AT3G16240; Glyma. 07g018000) by *P*. *indica*, suggesting that urea may be important in this symbiosis. Alternatively, *P*. *indica* may improve N uptake indirectly by stimulating rhizobial symbionts, as suggested by the increased rhizobial biomass:root biomass (Table 1) and upregulation of genes involved in biosynthesis of the flavonoid luteolin (Supplementary Table S5), which has been reported to regulate nodulation^113^, in *P*. *indica* colonized plants. However, we did not observe upregulation of ammonium transporters known to be involved in transfer of N from rhizobia to plants (Supplementary Table S5). *P*. *indica* also significantly improved shoot content of the beneficial nutrient iron, potentially through upregulation of two FER-like regulators of iron uptake (Glyma.13g322100 and Glyma.12g178500) and production of iron-scavenging compounds like scopoletin (Fig. 4 and Supplementary Table S5).

Induction of plant hormones by *P*. *indica* has been previously documented and is thought to play a role in its strong growth promoting and stress modulating effects^23,32^. Gibberellins, naturally occurring plant hormones that promote cell growth and stem elongation as well as aspects of flowering and fruiting^114–116^, have also been shown to have roles in primary root development, cell elongation^117,118^, and regulation of whole plant carbon metabolism^119^. While GA has been shown to increase root growth in some plants, it may also preferentially direct growth to the shoot, as observed in this study (Table 1). Our data showed upregulation of GASA14, (Supplementary Tables S5 and S7) a GA responsive gene shown to improve salt and ABA tolerance of transgenic *Arabidopsis* plants^120^. Additionally, a negative transcriptional regulator of GA biosynthesis (DDF1) was downregulated (Supplementary Table S5). Overexpression mutants of this gene (*ddf1*) showed decreased GA production, reduced height, and delayed flowering in *Arabidopsis*. The increased shoot biomass growth, faster flowering, and shorter time to seed production observed in our study could be due in part to downregulation of this or other negative regulators of GA.

The phytohormone ABA plays a crucial role in adaptive responses to environmental stresses, as higher ABA expression in response to drought and high salinity induces massive changes in gene expression resulting in stomatal closure^31,121^. An overexpression mutant of a homolog of an ABA responsive gene that was upregulated in our data (Supplementary Tables S5 and S7), ATbetaFruct4, showed higher levels in leaf stomatal guard cells and led to increased drought tolerance in *Arabidopsis*^80^. Transgenic *Arabidopsis* overexpressing GolS2, a homolog of another upregulated ABA responsive and drought-inducible gene involved in synthesis of galactinol (Supplementary Tables S5 and S7), showed greater drought tolerance due to higher intracellular osmolarity from galactinol and raffinose^122^. Other upregulated genes mapped to the pathways involved in biosynthesis of B-series fagopyritols and stachyose, compounds documented in both soybean and grasses^123^ to enhance seed desiccation tolerance during seed maturation (Supplementary Table S9). While these compounds have not previously been implicated in desiccation tolerance in vegetative parts of the plant, further research is needed to investigate their potential role in controlling osmotic stress.

Given *P*. *indica’s* documented ability to confer adaptation to abiotic stresses such as drought^20,124,125^ and salt stress^22,126,127^, the downregulation of heat shock and other proteins involved in heat acclimation was surprising. However, another recent whole transcriptome study of the response of *Arabidopsis* to both *P*. *indica* and the related sebacinoid fungus *Sebacina vermifera*, showed that several classes of heat shock proteins were consistently downregulated by both fungi^128^, suggesting this may represent a conserved response in plants to colonization by sebacinoid fungi. It is possible that *P*. *indica* may induce other mechanisms to increase tolerance to heat, high light, and hydrogen peroxide. We hypothesize that downregulation of expression of heat shock proteins in response to these abiotic stressors in *P*. *indica* plants compared to control plants experiencing the same conditions may indicate that the colonized plants are reducing the negative effects of abiotic stresses using other mechanisms and thus do not need to upregulate heat shock proteins. For example, the upregulation of homologs of a galactinol synthase (GolS2; AT1G56600; Glyma. 03g229800) and a sucrose vacuolar invertase (ATbetaFruct4; AT1G12240; Glyma.01g211000), which increase drought tolerance in *Arabidopsis* through increased intracellular osmolarity and regulation of stomatal opening, respectively, are possible mechanisms that may mitigate cellular stress and ROS production under drought stress and thus decrease expression of proteins involved in response to ROS.

During the biotrophic phase of colonization, *P*. *indica* has been shown to suppress root innate immunity in order to colonize host root tissue^33^, potentially through modulation of GA and SA mediated signaling^29^. In addition to GA, ABA also controls growth and proliferation of the fungus through suppression of plant innate immunity in barley^29^. Similarly, external exposure of *Arabidopsis* seedlings to ABA increased levels of colonization by *P*. *indica*^29^. ABA exposure also decreased expression of two MAMP responsive genes whose homologs in soybean were also downregulated in our data (Supplementary Tables S6 and S8)^31^. Recent work also suggests that effectors produced by *P*. *indica* mediate suppression of innate immunity to allow for colonization^129^.

In barley, after the initial biotrophic phase, *P*. *indica* induces PCD^10,11^, which is required for root colonization by this symbiont in barley. Our data suggest that PCD may also be required for colonization of soybean roots by *P*. *indica*. PCD induced by *P*. *indica*, however, has different characteristics from the typical hypersensitive response (HR) of plants to pathogens in that neither typical defense markers such as pathogenesis related proteins^130^ nor accumulation of ROS^29^ have been detected at high concentrations in colonized roots. Instead, transmission electron microscopy suggests that ultrastructural changes associated with endoplasmic reticulum (ER) stress, as well as a simultaneous suppression of the plant’s adaptive ER stress response (e.g. unfolded protein response pathway), lead to cell death^131^. In this study, genes belonging to several GO categories involved in the respiratory burst response and the typical incompatible HR of plants against pathogens were downregulated (Fig. 3B and Supplementary Table S8), while those involved in ER stress response and protein folding were upregulated (Fig. 3A and Supplementary Table S7). These results provide support for the hypothesis that *P*. *indica* induces PCD through an ER mediated mechanism distinct from HR.

## Conclusions

Our results demonstrate that colonization of soybean with *P*. *indica* induced shoot growth and biomass accumulation, leading to higher shoot: root ratios as well as earlier flowering and pod formation. In this study, the fungus also increased nutrient acquisition of key macronutrients, including N, P, and K, as well as of several micro and beneficial nutrients, especially Fe. Colonization by *P*. *indica* resulted in larger dry biomass of rhizobial nodules per root dry weight in colonized plants, suggesting a potential synergistic interaction of this fungal symbiont with rhizobia.

Transcriptomic analyses revealed that among the most highly upregulated genes and GO categories were those involved in lignin and cell wall synthesis, cell wall remodeling, and iron acquisition. In particular, genes (e.g. COMT) in the phenypropanoid pathway involved in monolignol biosynthesis, as well as several classes of oxidative enzymes such as laccases and peroxidases involved in polymerization of monolignol subunits in plant cell walls, were upregulated. Several genes involved in synthesis of plant secondary compounds, including aromatic compounds (1, 3, 5 trimethoxybenzene) and flavonoids (luteolin and, quercetin), both derived from the phenylpropanoid pathway, as well as furaneols and the volatile terpene linalool, were also upregulated. The upregulation of genes involved in biosynthesis of the flavonoid luteolin, which has been reported to regulate nodulation^113^, suggests a possible mechanism for the increased rhizobial biomass observed in the *P*. *indica* treatment. Synergistic interactions with rhizobia may be a potential mechanism for increased N uptake observed in *P*. *indica* colonized plants. We also demonstrate that *P*. *indica* induces increased expression of a plant PAP gene that is involved in solubilization and uptake of P. Other upregulated GO categories included those involved in hormone signaling. Our results support previous findings in other plant hosts that growth and abiotic stress responses induced during colonization by *P*. *indica* may be modulated by plant hormones. In particular, homologs of a number of *Arabidopsis* ABA responsive genes (GolS2 and ATbetaFruct4) with roles in response to drought stress were upregulated. Increased expression of genes involved in synthesis of other potential osmoregulatory compounds, B-series fagopyritols and stachyose, which have been shown to increase desiccation tolerance in seeds, was also observed.

Many downregulated genes belonged to GO categories involved in the plant defense response to fungal colonization. As is previous studies in Arabidopsis^33^, MAMP and PTI innate immunity pathways in soybean were suppressed in soybean roots colonized with *P*. *indica*. Similarly, suppression of the respiratory burst and some gene involved in HR in our data also support generalized suppression of the plant defense response to allow for colonization of roots by this symbiont. However, our results suggest that like in barley, a form of PCD is important for colonization of soybean roots. Results showed upregulation of genes in GO categories involved in response to ER stress and ER unfolded protein response, supporting microscopic findings that PCD involved in colonization by *P*. *indica* may not involve a typical HR response but is mediated by a distinct mechanism that involves ER stress. One of the more surprising results of this study was the pervasive downregulation of heat-shock proteins belonging to GO categories involved in response to heat, high light, hydrogen peroxide, and salinity stress. We hypothesize that *P*. *indica* may use other mechanisms to increase plant tolerance and decrease negative effects of abiotic stresses. Thus, the downregulation of heat shock proteins may reflect the ability of *P*. *indica* colonized plants to better tolerate or ameliorate these stresses without inducing oxidative stress to the plant.

Overall, this research sheds light on molecular mechanisms contributing to the increased growth, improved nutrient status, and stress tolerance of soybean colonized by *P*. *indica* and provides a foundation for exploring the tri-partite interactions between soybean, fungal, and rhizobial symbionts.

## Materials and Methods

### Growth of Plants and Fungus

Seeds of *G*. *max* Williams 82 were surface-sterilized and seeds of equal size were selected and pre-germinated. *P*. *indica* strain ATCC (204458) was propagated in potato dextrose broth as described previously^25^. Soil mix (Sunshine Professional Growing Mix) was autoclaved two times for 90 minutes with a 24-hour interval to sterilize. For inoculation of plants with *P*. *indica*, 2 g of mycelium were thoroughly mixed with 100 g of soil in peat pots before sowing. The control treatment contained no inoculum. Plants were grown in a Conviron growth chamber with temperatures of 22 °C at night and 24 °C during the day and a 16:8 hour light: dark cycle. Two different treatments including an uninoculated control and *P*. *indica* inoculated treatment were employed. Each treatment consisted of three biological replicate plants grown in separate peat pots. All the treatments were inoculated after 15 days of sowing with 10 mL of Optimize® LCO Promoter Technology® for soybean, Novozymes, according to manufacturer’s protocol. The inoculum contained 5 × 10^6^ spores/mL of *Bradyrhizobium japonicum*. Plants were harvested 60 days after sowing. Shoot length, fresh weight of aerial parts, and the number of pods were counted at harvest. The roots were thoroughly washed and fresh root weight was determined. The root volume was determined by water displacement using a measuring cylinder.

### Staining of Roots and Percent Colonization

Washed roots were cleaned by soaking in 10% KOH for 4 days, acidified with 1 N HCl for 5 minutes, and then stained with lacto-phenol cotton blue. The roots were observed under 630X with an optical light microscope (Olympus BH2, Leeds Precision Instrument, Inc.). Estimation of percent colonization was done using the grid line-intersect method^132^. Chlamydospores within roots were counted under the microscope and percent root colonization was calculated with the formula: % colonization = number of colonized roots × 100/number of roots observed^133,134^.

### Estimation of Macro, Micro and Beneficial Elements

The macroelements (N, P, K), microelements (Al, Mn, Ni, Zn, B, Cd, Cr, Cu) and other elements (Ca, Fe, Mg, Na) were estimated using Dumas method for total N^135,136^ and the ICP-dry ash method for other elements^137^ by nutrient analysis of shoot tissue. Plant nutrient testing was outsourced to the Research Analytical Laboratory, University of Minnesota.

### Statistical Analyses of Plant Growth Parameters and Nutrient Content

For analyses of plant growth parameters and nutrient content, the mean and standard deviation of three biological replicates, consisting of three individual plants grown in separate peat pots, were calculated. Statistical analyses of differences in plant growth responses and nutrient content across treatments were analyzed using a Dunnett’s multiple comparisons test. A p-value less than 0.05 was considered statistically significant. GraphPad Prism v6.04 was used for the analysis (GraphPad Software, La Jolla California USA, www.graphpad.com).

### RNA Isolation and Purification

Roots from the two biological replicate plants per treatment were randomly selected for RNA sequencing. For each biological replicate, three root sections of approximately 5 cm in length were randomly collected from three locations on each plant root system and were pooled for RNA extraction. Soybean root tissue samples were ground in liquid nitrogen using a mortar and pestle immediately after harvest and directly mixed with TRIzol^TM^ (Invitrogen) solution for extraction. Total RNA was isolated from the roots using the standard TRIzol^TM^ (Invitrogen) protocol including a chloroform extraction followed by precipitation in 0.1% 3 M NaAcetate pH 5 and 0.8 volumes cold isopropanol. Contaminating DNA was removed by treatment with DNAase A^138^ for 15 minutes at 37 °C. RNA was further purified and concentrated using the RNA cleanup method on a Plant RNeasy column (Qiagen). RNA quality was evaluated by gel electrophoresis, spectrophotometer, and Agilent 2100. Only samples with an RNA integrity number (RIN) >7.5 were used for sequencing. Total RNA was sent to the University of Minnesota Genomics Center for library prep using the Illumina TruSeq 2 RNA kit and adaptors. Samples were barcoded and sequenced using 100 bp single end reads on the Illumina Hi-Seq 2000.

### RNA-Seq Data Analysis

Data analysis packages within the Galaxy platform (www.galaxy.umn.edu) were used to analyze and perform quality control of the Illumina data, align sequenced reads to the *G*. *max* (Williams 82 a2.v1 downloaded from Phytozyme 10) reference genome and perform differential expression analyses. The Fastx Toolkit^139^ was used to assess the quality of the data. Trimmomatic^140^ was then used to remove any remaining Illumina adapters and to trim off 15 bases from the beginning of the reads and 10 bases at the end of the reads of poorer quality sequence to give a total read length of 75 bp. The average quality score for all reads was above 30. The reads were then aligned to the reference genome of Williams 82 using TopHat2^141^ and differential expression was performed with CuffDiff^142^ using the Williams 82 a2.v1 annotations. We used the geometric method for library normalization and a false discovery rate of 0.05. Genes with an FDR corrected p-value (q-value) less than 0.05 were considered differentially expressed. Functional annotation and *Arabidopsis* homologs were identified using SoyBase annotations.

### Validation of Gene Expression by qRT-PCR

To validate the expression of upregulated genes of *G*. *max*, cDNA was synthesized using approximately 0.3 μg of total RNA isolated from all three biological replicates of colonized and non-colonized roots samples. The cDNA was synthesized using a cDNA synthesis kit (Two-Step RT-PCR Kit from Affymetrix). Gene specific primers were designed with IDT-primer quest (http://www.idtdna.com/primerquest/home/index). Primer pairs used for gene expression analysis were designed using published cDNA sequences (https://www.soybase.org/) for *G*. *max* for selected genes (Glyma.13G191400, Glyma.05G188900, Glyma.11G213500, Glyma.08G076000, and Glyma.13G191400) (Supplementary Table S11). Primers previously tested for Elongation factor1b (Ef1b) gene of *G*. *max* was used as the housekeeping gene^143^. The relative expression of genes was quantified using qRT- PCR (Applied Biosystems 7500 Real-Time PCR System). To prepare the reaction mixture, 1.9 μl of template of a 1:5 dilution of prepared cDNA sample), 6.25 μl iTaq™ Universal SYBR® Green Supermix, and 0.5 μl of each gene specific primer pair were mixed with 3.35 μl of nuclease-free water to make a total volume of 12.5 μl. Thermal cycle conditions included hot start at 95 °C for 3 min followed by 40 cycles at 95 °C (30 s) and 58 °C (30 s). The melt curve was done at 95 °C (15 s), 58 °C (1 min), 95 °C (15 s) and 58 °C (15 s). All reactions were run with three technical triplicates for each of the two biological replicates. The ΔΔCT method was used to determine relative fold change in gene expression levels.

### Gene Ontology Analyses

A Gene Ontology (GO) enrichment analysis was performed on the SoyBase website (http://soybase.org/) to identify GO categories to which upregulated and downregulated genes belonged and to identify those that were over- or under-represented. In order to provide a broad overview of up and down regulated GO categories, both genes that were statistically enriched at a corrected p-value of 0.05 as well as those that were not significantly enriched but had a corrected p-value < 10 were displayed using the web server for REVIGO (http://revigo.irb.hr/) using an intermediate similarity cutoff of 0.7^59^. The resulting clusters were exported as a tree network and visualized in Cytoscape^61^. Clusters were color coded in Cytoscape according to their corrected p-value.

### Metabolic Pathway Analyses

The normalized expression levels of significantly up and down regulated genes for the control versus *P*. *indica* were mapped to the SoyCyc metabolic database in Soybase in order to identify metabolic pathways affected. All of the significantly upregulated or downregulated genes were displayed with a cutoff threshold of 0.05. The SoyCyc pathway database does not perform statistical analyses but provides a color coded scale of relative fold change of expression levels ranging from red (most upregulated) to blue (most down-regulated). *Arabidopsis* homologs of up and down regulated genes were also mapped to selected pathways in the KEGG pathway database to display up- or downregulated genes^144–146^.

### Data availability

Transcriptome raw Illumina reads are deposited in the short read archive at NCBI under submission number SUB3229927.

## Electronic supplementary material

Supplementary Tables S1-4 and S11

Supplementary Table S5

Supplementary Table S6

Supplementary Table S7

Supplementary Table S8

Supplementary Table S9

Supplementary Table S10
