# Supplementary material for: Transcriptional responses of soybean roots to colonization with the root endophytic fungus Piriformospora indica reveals altered phenylpropanoid and secondary metabolism
Source: Sci Rep. 2018 Jul 6;8:10227. doi: 10.1038/s41598-018-26809-3 (PMC6035220; doi:10.1038/s41598-018-26809-3)
Supplement: Supplementary file 1 — Supplementary Tables S1-4 and S11 [file 41598_2018_26809_MOESM1_ESM.pdf]

Supplementary Tables S1-4 and S11

Transcriptional responses of soybean roots to colonization with the root endophytic fungus *Piriformospora indica* reveals altered phenylpropanoid and secondary metabolism

Ruchika Bajaj, Yinyin Huang, Sebhat Gebrechristos, Brian Mikolajczyk, Heather Brown, Ram Prasad, Ajit Varma, Kathryn E. Bushley

Supplementary Table S1. Quantification of root colonization by *P. indica*.

	Percent Colonization by *P. indica* in soybean roots	
	Control	*P. indica*
Replicate 1	0	35
Replicate 2	0	49
Replicate 3	0	41
Average	0	41.66666667
Standard deviation	0	7.023769169

More than 100 fields of view of root sections randomly selected from each of three replicate plants per treatment were observed under the microscope after staining with Lactophenol Cotton Blue. The percentage colonization was calculated using the formula: *percent colonization = number of roots colonized/total number of roots observed *100*.

Quantification of root colonization by *P. indica*. A) Sample of images from control roots showing no fungal colonization, B) Sample of images from *P. indica* treated roots showing stained hyphae and pear-shaped chlamydospores (blue arrows).

A) Sample of images from control roots showing no fungal colonization.

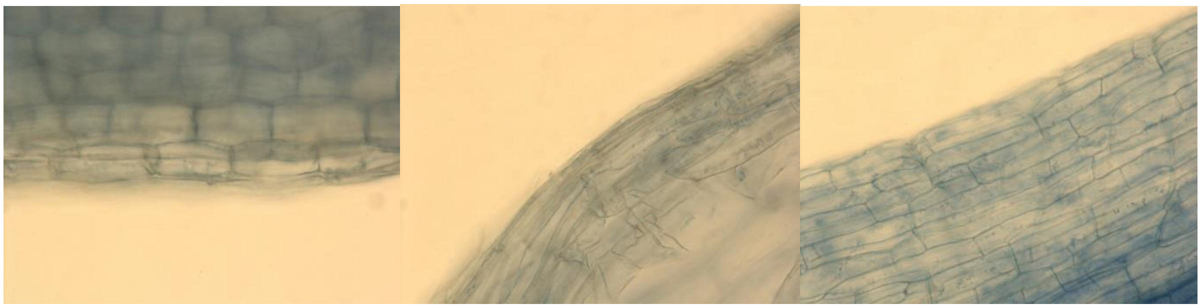

B) Sample of images from *P. indica* treated roots showing stained hyphae and pear-shaped chlamydospores (blue arrows).

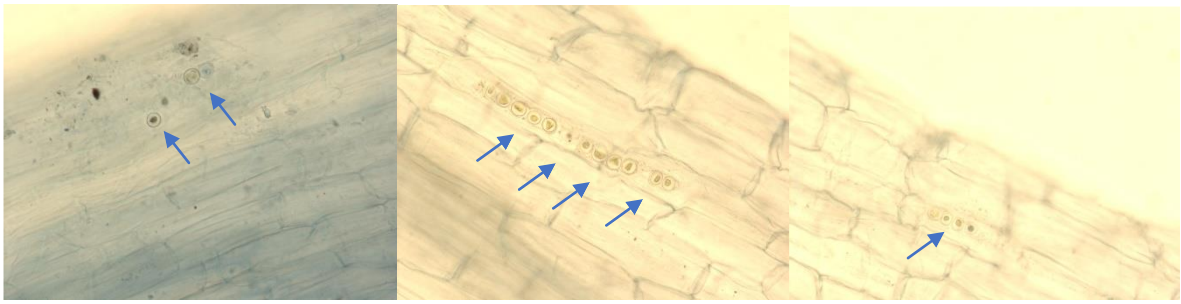

Supplementary Table S2. Effect of *P. indica* on concentration of microelements. The mean and standard deviation were calculated from three plant biological replicates. An asterisk (*) indicates significance at p = 0.05 using Duncan's multiple comparison test between the control and *P. indica* treatment.

Elements	**Control**	***P. indica***	**% increase of *P. indica* over control**
**Micro-elements**			
Aluminium (mg/kg)	7.42 ± 1.66	15.59 ± 2.58*	110.15
Manganese (mg/kg)	170.21± 4.01	213.29± 13.22*	25.31
Nickel (mg/kg)	0.42 ± 0.05	0.62 ± 0.15*	46.98
Zinc (mg/kg)	22.46 ± 0.59	24.48 ± 0.64*	8.97
Boron (mg/kg)	24.41 ± 0.02	21.34 ± 1.23*	-12.56
Cadmium (mg/kg)	0.13 ± 0.05	0.05 ± 0.03*	-92.48
Chromium (mg/kg)	0.42 ± 0.01	0.38 ± 0.03*	-10
Copper (mg/kg)	4.30 ± 0.27	3.64 ± 0.74*	-15.39
**Beneficial Elements**			
Calcium (mg/kg)	11696.67± 336.02	13476.3± 1314.31*	15.25
Iron (mg/kg)	65.23 ± 1.01	91.59 ± 6.467*	40.40
Magnesium (mg/kg)	3379.97 ± 32.02	3899.17 ± 232.95*	15.36
Sodium(mg/kg)	284.49 ± 11.62	306.96 ± 29.42*	7.90

Supplementary Table S3. Percentage of reads aligned to Williams 82 *G. max* genome.

**Percentage of reads aligned to *G. max***			
**Sample**	No. of Reads Aligned	Total no. of Reads	% of Total Reads Aligned
**Control 1**	11316472	12059044	93.84
**Control 2**	13342899	14144169	94.33
***P. indica* 1**	10584811	11209774	94.42
***P. indica* 2**	11570287	12239803	94.53

Supplementary Table S4: qRT-PCR validation of differential expression.

The cDNA library was prepared using approximately .5µg total RNA and cDNA was diluted 1:5 for use as template for qRT-PCR. The average CT values and relative fold change calculated from the average of three biological replicates for the selected genes which were upregulated in our data is given below:

Primer Pair	Gene ID	CT of *P. indica* Trt	CT of Control	Relative fold Change	RNA-Seq Fold Change
GFH32	Glyma.01G211000	32.19	32.89	2.44	2.11
RING	Glyma.05G188900	28.78	28.89	1.78	2.10
TPX2	Glyma.11G213500	30.10	30.92	2.63	2.23
VIT	Glyma.08G076000	28.54	29.46	3.10	2.71
STA2	Glyma.13G191400	32.41	31.10	2.56	2.31

Supplementary Table S11: Primers used for qRT-PCR validation of differential expression.

Primers were designed and synthesized with the help of IDT-primer quest (http://www.idtdna.com/primerquest/home/index). The elongation factor 1b (Elf1b) gene of *G. max* was used as a housekeeping to normalize experimental gene expression [1]. Primer pairs used for gene expression analysis were designed according to published cDNA sequences (https://www.soybase.org/) for *G. max* (Williams 82 a2.v1).

Pimers:

Gene ID	Forward Primer	Reverse Primer
Elf1b	GTTGAAAAGCCA GGGGACA	TCTTACCCCTTGA GCGTGG
Glyma.07G014500	GGTCCTTGTTTTCTTCGTTGC	ATAGCCAGTGTTCATTCGGAG
Glyma.05G188900	GCCCTCAAGACTTTTCCTACTG	TGCGAACCTTATCACCATTGG
Glyma.11G213500	CAAACTCCACACCCAAGAAAG	AGCAAACGAACCTCTTCCTC
Glyma.13G191400	GGCCACAATTCCAAAATCAGG	GAAAGGCACAAGTTCATGAGG
Glyma.15G014500	GTTCATTCTCAGTGCTTTCGTC	CACGATGACTCCTAACGGTTC

1. Jian, B., et al., *Validation of internal control for gene expression study in soybean by quantitative real-time PCR.* BMC Molecular Biology, 2008. **9**: p. 59-59.
